# Case Report: Hypertrophic cardiomyopathy meets left ventricular hypertrabeculation: from mixed cardiomyopathy to hypertrophic cardiomyopathy with hypertrabeculation

**DOI:** 10.3389/fcvm.2025.1708374

**Published:** 2025-11-26

**Authors:** Yang Li, Yang Liu, Xiaofei Zheng, Lijuan Chen, Qiming Dai, Genshan Ma

**Affiliations:** 1 Department of Cardiology, School of Medicine, Zhongda Hospital, Southeast University, Nanjing, China; 2 Department of Radiology, School of Medicine, Zhongda Hospital, Southeast University, Nanjing, China

**Keywords:** left ventricular non-compaction/hypertrabeculation, hypertrophic cardiomyopathy, heart failure, cardiac MR, cardiomyopathy

## Abstract

This case report describes a young male patient admitted due to worsening of heart failure with recurrent embolic events. He had previously been diagnosed with hypertrophic cardiomyopathy (HCM) and left ventricular systolic dysfunction at other hospitals and was treated irregularly with medications including a beta-blocker and diuretics. On this admission, echocardiography and cardiac MR examination revealed left ventricular myocardial non-compaction combined with HCM. The coexistence of left ventricular hypertrophy and hypertrabeculation is rare, and the diagnosis has been updated according to the newly issued European Society of Cardiology guidelines. The present report describes the clinical features, therapy, and outcome of this disease.

## 1 Introduction

Previous cases demonstrated that both hypertrophic cardiomyopathy (HCM) and left ventricular non-compaction (LVNC) can rarely co-exist in the same patient (1–4). The mixed phenotype (HCM and LVNC) is associated with a more severe clinical course of the disease and worse cardiovascular complications (5). This case report describes a young male patient with HCM and hypertrabeculation.

## 2 Case presentation

A 39-year-old male patient was admitted to our hospital in April 2022 due to repeated chest tightness and shortness of breath for 5 years, aggravated for 20 days. He presented previous echocardiography examinations in other hospitals at admission, all indicated non-obstructive HCM, reduced left ventricular ejection fraction (the lowest was 12%), and enlarged left ventricle. He was repeatedly hospitalized and treated with irregular oral administration of a beta-blocker and diuretics before this admission. The previous electrocardiogram featured with sinus rhythm, frequent ventricular premature beats with short bursts of ventricular tachycardia, intraventricular conduction block, and occasional atrial premature beats. He was diagnosed with acute renal infarction in December 2020, acute hemorrhagic cerebral infarction in June 2021, and right middle cerebral artery occlusion and left middle cerebral artery inferior trunk occlusion in January 2022. He underwent emergency thrombectomy for an embolic event in 2022 and irregularly took oral warfarin after the first embolic event.

He was an ex-smoker with more than 10 years of smoking history, smoking approximately 10 cigarettes per day. He denied a history of hypertension, diabetes, and excessive alcohol exposure. There was no similar case in his family.

Physical examination at admission: a few moist rales were heard in the right lower lung, heart rate was 66 beats/min, a systolic murmur of grade 2/6 was heard in the apex of the heart, and there was no edema in either lower limb. Troponin I was 0.51 ng/mL, NT-pro BNP was 17,500 pg/mL, and D-dimer was 932 µg/L.

Electrocardiogram: sinus rhythm, high voltage in left ventricle, high voltage in right ventricle, premature ventricular beats, intraventricular conduction block, short P–R interval, abnormal ST segment and T wave changes. Chest CT scan showed the following: (1) interstitial pulmonary edema in both lungs, scattered cord foci in both lungs; (2) enlarged heart shadow, pericardial effusion, small amount of pleural effusion on both sides, and incomplete expansion of adjacent lung tissue, especially on the right side. Coronary angiography showed no obvious stenosis of the coronary artery.

**Figure 1:**
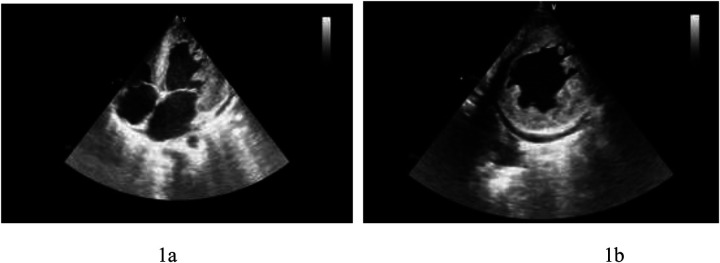
**(a)** Left ventricular apical four-chamber view showing left ventricular thickening and hypertrabeculation. **(b)** Left ventricular short-axis view presenting left ventricular thickening and hypertrabeculation.

Echocardiography: myocardial involvement [interventricular septum (IVS): 2.16 cm, left ventricular posterior wall (LVPW): 2.39 cm], left ventricular enlargement (LV: 6.6 cm), mild to moderate mitral regurgitation, mild tricuspid regurgitation, mild pulmonary hypertension, significantly reduced left ventricular function (EF: 20%), and a small amount of pericardial effusion. Significant trabeculae in the middle segment of the left ventricular free wall and the apical segment of the left ventricular wall were visualized, showing a “honeycomb-like” change. The ratio of the thickness of the non-compacted myocardium (∼16 mm) to the thickness of the compacted myocardium (∼8 mm) at the end of contraction was ∼2:1. Color Doppler ultrasound detected low-speed blood flow in the gaps between the myocardial recesses that communicated with the left heart cavity (Figure 1).

**Figure 2:**
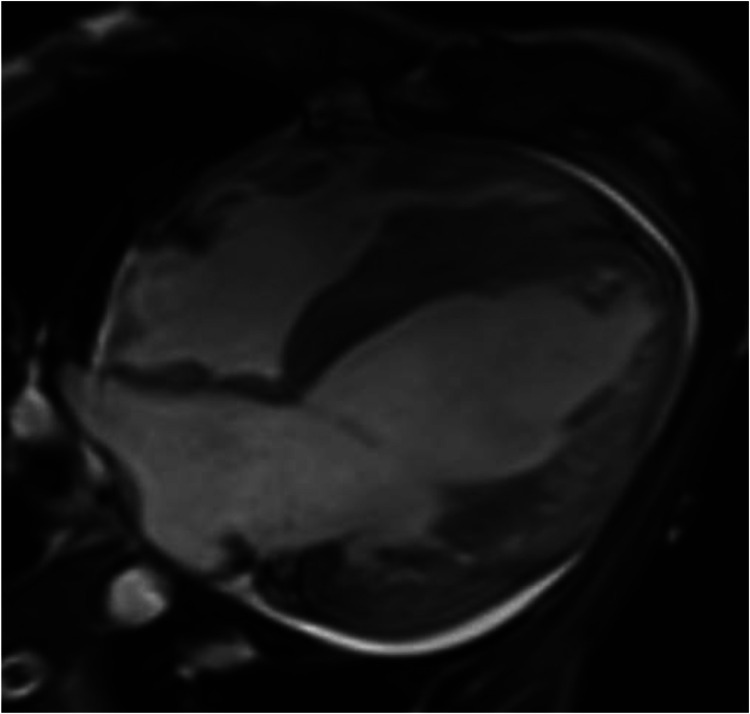
CMR four-chamber heart long axis: left ventricular dilatation, thinning of left ventricular free wall and apex, with increased and disordered trabeculae, presenting a spongy appearance.

Cardiac magnetic resonance (CMR) plain scan + enhanced scan showed the following: (1) left ventricular dilatation with decreased systolic function (EF 19%) thinning of the left ventricular free wall and apex, weakened movement, and increased and disordered trabeculae, considering LVNC; late enhancement of the left ventricular anterior wall, lateral wall, inferior wall, and apex, suggesting myocardial fibrosis or scar; (2) thickening of the left ventricular septum and right ventricular myocardium, with a high possibility of HCM; and (3) pericardial effusion (Figure 2).

The patient was diagnosed as non-obstructive HCM combined with LVNC and received recommended medication as recommended by the 2021 European Society of Cardiology (ESC) guidelines for the diagnosis and treatment of acute and chronic heart failure (6) and the 2014 ESC guidelines on diagnosis and management of HCM (7). The patient was discharged after symptom relief. The patient was at high risk of sudden cardiac death. Medication at discharge was as follows: metoprolol 47.5 mg qd, canagliflozin 100 mg qd, sacubitril/valsartan 50 mg bid, spironolactone 20 mg bid, furosemide 20 mg qd, and rivaroxaban 15 mg qd. Despite these medications, the patient died of sudden cardiac death 6 months later.

## 3 Discussion

LVNC and HCM are cardiomyopathies with distinct clinical presentations but may present common genetic mutations (4). Coexistence of HCM and LVNC is rare (8). Before the 2023 ESC guidelines for the management of cardiomyopathies, developed by the task force on the management of cardiomyopathies of the ESC (9), patient with this clinical phenotype was often diagnosed as HCM and LVNC. Now, this disease phenotype is named as HCM with hypertrabeculation (1).

**Figure 3:**
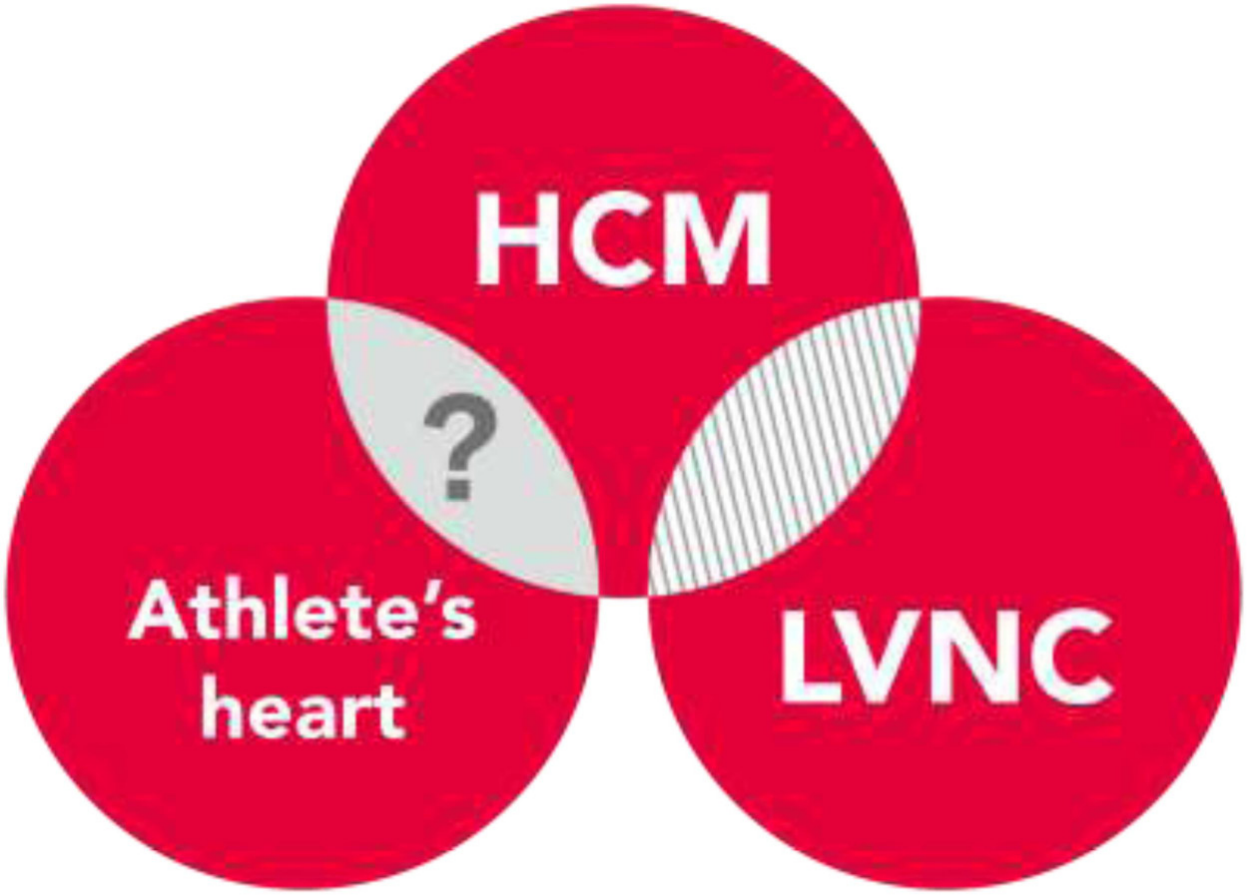
Differentiating an athlete's heart from HCM.

HCM is often manifested as hypertrophy of the ventricular septum, while this case presented both hypertrophy and hypertrabeculation. Detraining and CMR can be used to differentiate cardiomyopathies from athlete's heart (10–12) (Figure 3). Despite standard medication at that time, and before the era of cardiac myosin inhibitors (13), the patient suffered sudden cardiac death within 6 months after discharge. Neither genetic studies nor an autopsy were performed on the patient. These missing data served as a significant limitation of the current report for the understanding of the underlying pathogenetic background of this patient.

It remains unknown if this patient with non-obstructive HCM might benefit from the cardiac myosin inhibitor therapy on top of the medication received. The cause of death might be related to malignant ventricular arrhythmias. It remained unknown if this patient might benefit from implantable cardioverter defibrillator (ICD) therapy or arrhythmia ablation. Nowadays, therapy options for HCM have been significantly expanded (14), especially for obstructive HCM. It is expected that more therapeutic options might also be available for patients with non-obstructive HCM and hypertrabeculation.

## Data Availability

The original contributions presented in the study are included in the article/Supplementary Material, further inquiries can be directed to the corresponding author.
